# NLRP3 and beyond: inflammasomes as central cellular hub and emerging therapeutic target in inflammation and disease

**DOI:** 10.3389/fimmu.2025.1624770

**Published:** 2025-09-01

**Authors:** María Virginia Pinzón-Fernández, Jhan S. Saavedra-Torres, Nelson Adolfo López Garzón, Jessica S. Pachon-Bueno, Francisco Javier Tamayo-Giraldo, María Camila Rojas Gomez, Marlon Arias-Intriago, Alice Gaibor-Pazmiño, Andrés López-Cortés, Juan S. Izquierdo-Condoy

**Affiliations:** ^1^ Grupo de Investigación en Salud (GIS), Universidad del Cauca, Popayán, Colombia; ^2^ Facultad de Salud, Universidad Santiago de Cali, Cali, Colombia; ^3^ Facultad de Medicina, Universidad del Valle, Cali, Colombia; ^4^ Departamento de Clínicas Médicas, Universidad Javeriana, Cali, Colombia; ^5^ Facultad de Medicina, Universidad del Rosario, Bogotá, Colombia; ^6^ One Health Research Group, Universidad de las Américas, Quito, Ecuador; ^7^ Cancer Research Group (CRG), Universidad de Las Américas, Quito, Ecuador

**Keywords:** inflammasome, pyroptosis, caspase 1, pattern recognition receptors (PRRs), interleukin-1 beta (IL-1β), programmed cell death, NLRP3

## Abstract

The NLRP3 inflammasome is a key cytosolic sensor in the innate immune system, activated by diverse danger signals such as metabolic stress, infections, and structural cellular disruptions. Its activation leads to the maturation of IL-1β and IL-18 and induces pyroptosis through gasdermin D cleavage. Multiple regulatory mechanisms modulate NLRP3 activation, including BRCC3-mediated deubiquitination, lysine carbamylation, intracellular trafficking to the microtubule-organizing center, and endolysosomal localization via PI4P. Dysregulation of these checkpoints contributes to inflammatory, neurodegenerative, hepatic, metabolic, and infectious diseases. Beyond pathogen defense, inflammasomes influence tissue regeneration, cell death pathways, and sterile inflammation, highlighting their role as integrative immune hubs. Alternative inflammatory pathways involving gasdermin E and caspase-8/3 enable persistent cytokine release in the absence of gasdermin D, revealing redundant effector arms within the inflammasome network. Structural triggers such as potassium efflux and intracellular transport disruptions lower the threshold for inflammasome assembly, while hypoxic conditions link its activation to immunometabolic imbalance. Aggresome-like mechanisms further reflect a convergence between proteostasis and inflammation. While NLRP3 remains the most extensively characterized, other inflammasomes—including NLRP1 in epithelial ribotoxic stress, CARD8 in HIV-1 protease sensing, and AIM2/IFI16 in viral and DNA sensing—highlight the diversity of inflammasome signaling in tissue- and pathogen-specific contexts. Small molecules such as MCC950, thiolutin, HDAC6 inhibitors, and CuET have demonstrated efficacy in preclinical models by selectively modulating inflammasome components or their regulatory pathways. Novel strategies such as carbamylation-mediated suppression and disruption of endocytic dynamics offer additional therapeutic entry points. A deeper understanding of inflammasome biology is essential for advancing precision immunotherapy in inflammatory and infectious diseases.

## 1 Introduction

The NLRP3 inflammasome represents an essential component of the innate immune system, serving as a molecular platform that detects diverse danger signals including pathogen-associated molecules (e.g., LPS), cellular stress markers (e.g., ATP), and metabolic crystals (e.g., urate) (1, 2). This multiprotein complex assembles primarily in myeloid cells, where it orchestrates caspase-1–mediated maturation of IL-1β and IL-18, while inducing pyroptotic cell death to amplify inflammatory signaling (3). Although this pathway plays a protective role during acute infections, sustained NLRP3 activation contributes to chronic tissue damage and systemic inflammation (2). NLRP3 belongs to the NOD-like receptor (NLR) family, which also includes NLRP1, another inflammasome-forming sensor activated by distinct stimuli such as pathogen proteases and toxins (4, 5).

Recent research highlights the delicate balance governing NLRP3 activity, revealing its complex regulatory mechanisms. This essential immune component displays dual functionality: protective in acute responses but potentially harmful when chronically activated. For instance, in Parkinson’s disease (PD), α-synuclein aggregates disrupt autophagy and ubiquitination pathways, leading to persistent inflammasome activation and dopaminergic neuron degeneration (1). In addition to protein aggregates, other sterile stimuli—such as environmental particles, ion fluxes, and mitochondrial damage—also converge to activate NLRP3, reinforcing its central role in innate immunity (2, 3). These findings underscore the inflammasome’s paradoxical nature: while its transient activation is crucial for acute immune defense, chronic activation drives sustained inflammatory pathologies. Therapeutic strategies targeting specific regulatory nodes, such as deubiquitinating enzymes (3), offer promising avenues for selective inflammasome control. Recent studies over the past three years have explored additional mechanisms—including post-translational modifications (e.g., SUMOylation), metabolic rewiring, and phase separation—that further modulate inflammasome assembly and activity (6–8). These advances are reshaping our understanding of NLRP3 as a finely tuned regulatory complex rather than a binary switch.

Translating these discoveries into clinical practice presents significant challenges, particularly in balancing therapeutic efficacy with potential adverse effects. While corticosteroids remain the standard treatment for acute inflammatory conditions such as sepsis, their broad immunosuppressive action often undermines clinical benefits by impairing essential immune responses (2). Nonetheless, considerable progress has been made in developing pharmacological interventions that regulate NLRP3 through multiple critical pathways. Researchers have identified compounds that restore autophagic flux to counteract excessive inflammasome activation, as well as precise inhibitors targeting NLRP3 deubiquitination processes (3, 9). Notably, newer agents such as MCC950 analogues, INF39, and dapansutrile (OLT1177) have shown promise in preclinical and clinical trials, although limitations such as hepatotoxicity or low selectivity have hindered broader clinical application (10, 11). These challenges emphasize the need to contextualize inflammasome inhibition within disease-specific frameworks and to refine drug design for optimal efficacy and safety.

Particularly promising are novel therapeutic agents designed to selectively block NLRP3 assembly while preserving its essential immune surveillance functions. Such advances mark important progress in our capacity to therapeutically modulate inflammasome activity without compromising host defenses. A comprehensive understanding of the regulatory mechanisms governing inflammasome activation is critical for developing next-generation therapies that preserve its protective roles in host defense while mitigating its pathological effects in chronic inflammatory states (1–3).

## 2 Molecular regulation of the NLRP3 inflammasome

### 2.1 Post-translational modifications: ubiquitination and deubiquitination

One of the key mechanisms regulating the NLRP3 inflammasome involves post-translational modifications. Among these, polyubiquitination acts as a molecular brake that maintains NLRP3 in an inactive resting state. This process entails the addition of ubiquitin chains to specific lysine residues, thereby preventing its oligomerization and subsequent activation. To initiate its inflammatory signaling, NLRP3 must be deubiquitinated, enabling it to adopt an active conformation capable of interacting with the adaptor protein ASC and procaspase-1 (2, 9, 12).

**Figure 1:**
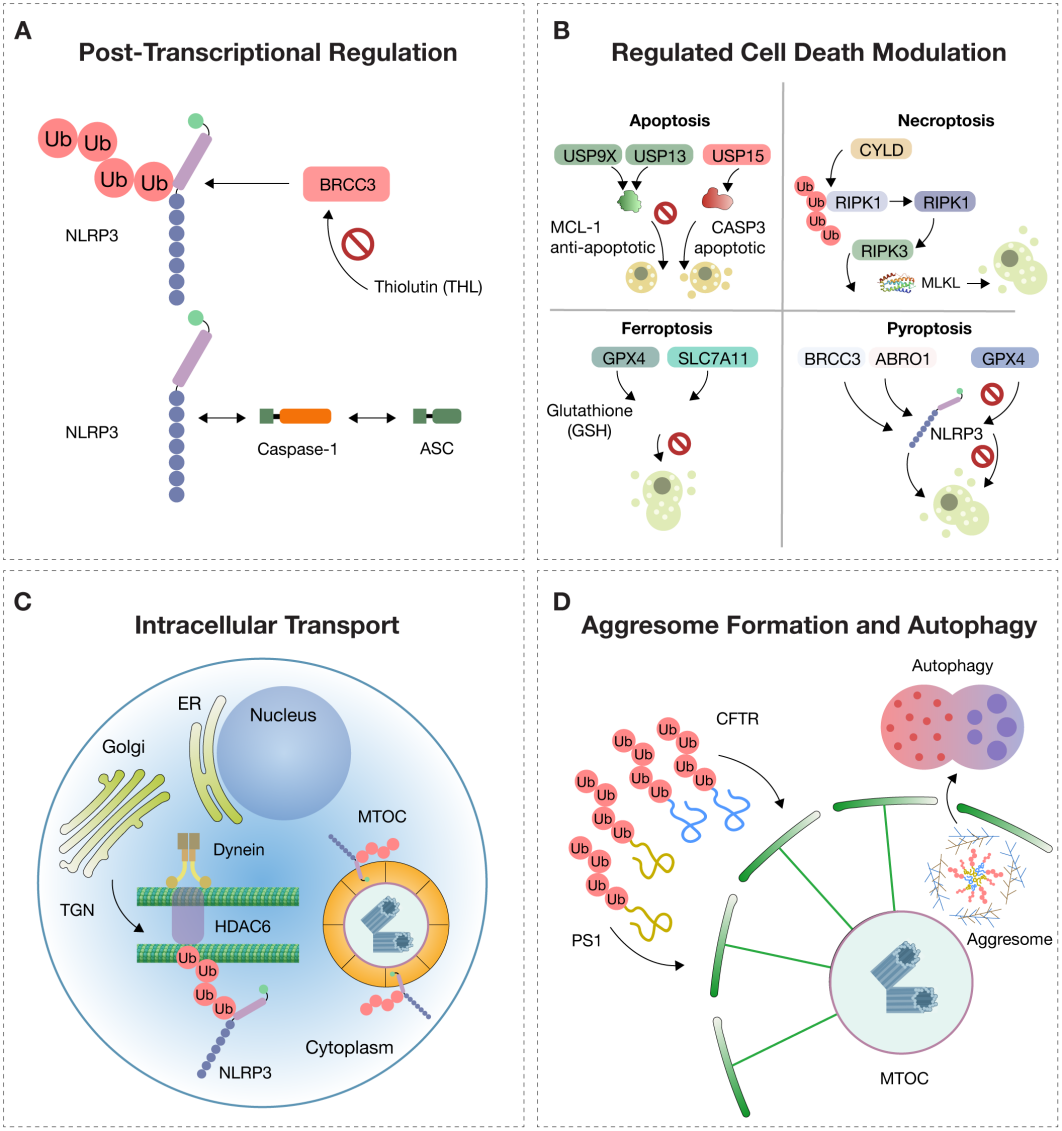
Molecular Regulation of NLRP3. The activation of NLRP3 is tightly controlled by multiple intracellular mechanisms. **(A)** Post-translational modifications: In the resting state, NLRP3 (NOD-, LRR-, and pyrin domain-containing protein 3) is polyubiquitinated, preventing its activation. The deubiquitinase BRCC3, part of the BRISC (BRCC36 isopeptidase complex), removes ubiquitin chains and enables NLRP3 to oligomerize with ASC (apoptosis-associated speck-like protein containing a CARD) and pro-caspase-1. Thiolutin (THL) inhibits BRCC3, blocking NLRP3 activation. **(B)** Deubiquitination regulates cell death pathways: USP9X and USP13 stabilize the anti-apoptotic protein MCL-1, while USP15 promotes apoptosis via procaspase-3 (CASP3); CYLD promotes necroptosis through activation of RIPK3 and MLKL; GPX4 and SLC7A11 prevent ferroptosis by maintaining glutathione (GSH)-dependent redox balance; BRCC3 and ABRO1 promote NLRP3-dependent pyroptosis, while USP5, CYLD, and TNFAIP3 inhibit it. **(C)** Intracellular trafficking: NLRP3 is trafficked from the trans-Golgi network (TGN) to the microtubule-organizing center (MTOC) along microtubules via dynein and HDAC6 (histone deacetylase 6), which recognizes ubiquitinated cargo and facilitates inflammasome assembly. **(D)** Aggresome dynamics: Misfolded proteins such as CFTR (cystic fibrosis transmembrane conductance regulator) and PS1 (presenilin-1), resistant to proteasomal degradation, accumulate and are transported to the MTOC, forming perinuclear aggresomes surrounded by vimentin filaments. These structures are cleared by selective autophagy (aggrephagy) to preserve proteostasis.

Deubiquitination thus emerges as an essential and tightly regulated step. This process is catalyzed by the BRISC (BRCC36 isopeptidase complex) enzymatic complex, whose active subunit BRCC3 removes ubiquitin chains and allows the functional assembly of the inflammasome. Through this action, BRCC3 enables NLRP3 to transition from a latent to a pro-inflammatory state, resulting in an effective immune response (2, 9, 12) (**Figure 1A**).

However, in autoinflammatory, neurodegenerative, metabolic, and cardiovascular diseases, excessive activation of this pathway has been observed and is associated with progressive tissue damage (2, 9, 12).

Given this evidence, the pharmacological inhibition of NLRP3 deubiquitination has emerged as a promising therapeutic strategy. Recent studies have identified thiolutin (THL) as a potent inhibitor of BRCC3, a JAMM-domain metalloprotease responsible for removing ubiquitin moieties from NLRP3. THL, a zinc chelator, inhibits BRCC3 activity at nanomolar concentrations. By targeting BRCC3 rather than NLRP3 directly, THL impairs the deubiquitination step required for NLRP3 activation. As a result, it effectively prevents inflammasome assembly and downstream inflammatory responses, even in the presence of canonical stimuli such as LPS or crystalline activators. This indirect mechanism underscores the therapeutic potential of targeting upstream regulatory nodes in the inflammasome activation pathway (13–15).

Additionally, THL has been shown to inhibit not only the canonical but also the non-canonical and alternative inflammasome pathways, highlighting the robustness and comprehensiveness of its action. The canonical pathway is triggered by stimuli such as toxins, crystals, or ATP, which activate caspase-1, leading to the processing of IL-1β and IL-18. The non-canonical pathway involves the intracellular detection of LPS by caspase-11 (in mice) or caspase-4/5 (in humans), indirectly activating NLRP3 (2, 9, 12).

Meanwhile, the alternative pathway is activated by LPS through Toll-like receptors (TLRs), particularly TLR4, without requiring cell death, resulting in a more gradual but sustained inflammatory response. The ability of THL to interfere with all three pathways underscores its therapeutic potential in complex inflammatory conditions (2, 9, 12).

THL has also demonstrated efficacy against constitutively active NLRP3 mutations, such as those seen in cryopyrin-associated periodic syndromes (CAPS). Its potential applications thus extend beyond acquired inflammatory states to include genetic autoinflammatory diseases (2, 9, 12).

Moreover, a natural derivative of thiolutin known as holomycin has been developed, showing superior inhibitory potency and reduced toxicity, enhancing the feasibility of translating this strategy into clinical use. In murine models, both THL and holomycin have shown significant benefits in diseases such as experimental autoimmune encephalomyelitis, LPS-induced sepsis, monosodium urate-induced peritonitis, and non-alcoholic fatty liver disease induced by a methionine- and choline-deficient diet (9).

From a pathophysiological perspective, these findings highlight how a single structural modification—deubiquitination—can modulate an entire inflammatory axis, making this step a strategic control point (2, 12).

### 2.2 Deubiquitinases in cell death pathways

Regulated cell death (RCD) is a dynamic process governed by specific molecular signals aimed at maintaining tissue homeostasis or eliminating damaged, infected, or senescent cells. This biological function is deeply influenced by post-translational modifications, among which ubiquitination and its reversal by deubiquitinases (DUBs) serve as decisive regulators of cell fate. By removing ubiquitin chains from target proteins, DUBs directly modulate apoptotic, necroptotic, pyroptotic, and ferroptotic pathways, making them essential control points in both normal physiology and disease (9, 12) (**Figure 1B**).

Pathophysiologically, ubiquitination serves as a tagging mechanism that controls protein stability, localization, and function. DUBs dismantle these tags, restoring or prolonging protein function. This dynamic balance involves enzymatic cascades comprising E1, E2, and E3 enzymes, while DUBs are categorized into seven evolutionarily conserved families, including USP, OTU, and JAMM, among others (16, 17).

In apoptosis, DUBs regulate key components of both the intrinsic and extrinsic pathways. For instance, USP8 and USP27X stabilize CFLAR, a potent inhibitor of caspase-8, while USP9X and USP13 maintain MCL1, a critical anti-apoptotic mitochondrial protein. Conversely, DUBs such as USP15 stabilize effector caspases like CASP3, facilitating their activation. This dual functionality enables DUBs to either promote or inhibit apoptosis depending on the context and cellular microenvironment (16, 17).

In necroptosis, a programmed form of death with a necrotic phenotype, the activation of RIPK1 and RIPK3 is tightly controlled by ubiquitination. The DUB CYLD promotes necroptosis by removing K63/M1 ubiquitin chains from RIPK1, facilitating ripoptosome formation. Similarly, OTULIN and USP22 modulate necroptosis by regulating RIPK3 polyubiquitination levels, demonstrating how DUB specificity can dictate the balance between controlled inflammation and destructive necrosis (16, 17) (**Figure 1B**).

In ferroptosis, an iron- and lipid peroxidation-dependent cell death pathway, DUBs like USP35 stabilize iron transporters such as SLC40A1, preventing toxic iron accumulation. Others, including USP11 and OTUB1, regulate antioxidant defenses by modulating SLC7A11 and GPX4, which are crucial for counteracting oxidative stress. Additionally, selective autophagy-induced ferroptosis is controlled by DUBs targeting BECN1 (e.g., USP14) or antioxidant transcription factors like NFE2L2, stabilized by USP11 (16, 17) (**Figure 1B**).

In pyroptosis, which is defined by inflammatory activation, gasdermin cleavage, and membrane pore formation, DUBs play central roles in controlling NLRP3 inflammasome activity. BRCC3 and ABRAXAS2 promote inflammasome activation through NLRP3 deubiquitination, while CYLD, TNFAIP3, and USP5 inhibit activation by promoting degradation or disrupting ASC interaction. Other DUBs such as UCHL5 can induce pyroptosis in response to specific bacterial stimuli, demonstrating the immune system’s plasticity in host defense (16, 17) (**Figure 1B**).

### 2.3 Intracellular transport and subcellular trafficking of NLRP3

Within immune cells, inflammasome assembly is not a random process. For NLRP3 or pyrin inflammasomes to become fully activated and trigger an inflammatory response, their components must be trafficked to a specific cellular location: the microtubule-organizing center (MTOC). This site serves as a convergence point for danger signals, activation of caspase-1, and the subsequent release of IL-1β and IL-18. However, to reach this site, the inflammasome must be transported from the trans-Golgi network (TGN), where it accumulates following inflammatory stimulation (16, 17) (**Figure 1C**).

This trafficking relies on a finely coordinated system involving microtubules, the dynein motor complex, and the adaptor protein HDAC6. HDAC6 not only serves as a transport facilitator but also recognizes ubiquitinated NLRP3 aggregates and directs them to the MTOC, where inflammasome assembly can proceed. Notably, this mechanism parallels cellular pathways used for managing damaged proteins through aggresome formation and autophagic clearance—illustrating how shared cellular machinery is utilized for both defense and proteostasis (16, 17).

From a clinical perspective, this discovery reshapes our understanding of inflammation at the cellular level. The requirement for NLRP3 transport to the MTOC introduces a novel therapeutic checkpoint: HDAC6. Inhibiting HDAC6 prevents inflammasome activation even in the presence of inflammatory stimuli, which is especially relevant in diseases involving chronic NLRP3 activation such as gout, hereditary autoinflammatory syndromes, and neurodegenerative disorders. Instead of global immunosuppression, targeted disruption of trafficking offers a precise way to inhibit inflammation at its structural origin (16, 17).

Additionally, the colocalization of the NLRP3 inflammasome with autophagy markers at the MTOC suggests that cells may attempt to regulate inflammatory activation via the autophagic machinery. This opens the possibility of using autophagy as a therapeutic pathway to selectively degrade inflammasome complexes—controlling inflammation while preserving immune competence (16, 17).

### 2.4 Aggresome formation and immunological implications

Aggresomes are specialized perinuclear structures that sequester misfolded or undegradable proteins, functioning as a protective response to proteotoxic stress. This compartmentalization prevents the accumulation of toxic protein aggregates and facilitates their clearance via autophagy. In contrast, the inflammasome is a multiprotein complex that detects signals of damage or infection, activating caspase-1 and inducing inflammation through IL-1β, IL-18, and pyroptosis. While aggresomes are closely associated with proteostasis and neurodegenerative diseases, inflammasomes are central to innate immunity and inflammatory disorders. Both represent adaptive cellular responses to internal threats but operate via distinct pathways and have different pathophysiological consequences (9, 16–18).

The aggresome is an organized cytoplasmic structure that constitutes a cellular adaptive mechanism against proteotoxic stress caused by the accumulation of misfolded, insoluble, and undegradable proteins through the ubiquitin–proteasome system. It forms when the production of defective proteins—due to mutations, cellular stress, or failure in protein folding—exceeds the degradative capacity of the proteasome. In these conditions, misfolded proteins are polyubiquitinated but cannot be efficiently degraded, leading to their aggregation and retrograde transport along microtubules to the MTOC. There, they concentrate in a pericentriolar inclusion stabilized by a network of intermediate filaments, primarily vimentin (16, 18–20) (**Figure 1D**).

These membrane-free inclusions consist of aggregates of integral membrane proteins such as the cystic fibrosis transmembrane conductance regulator (CFTR) or presenilin-1 (PS1). They typically appear as electron-dense particles of 60–80 nm, enriched in ubiquitin and surrounded by reorganized vimentin filaments forming a structural cage. Aggresome formation requires an intact microtubule cytoskeleton, and once formed, the structure remains stable and persists even after removal of the stressor (16, 18–20) (**Figure 1D**).

This cellular process serves a dual function. Initially, it acts as a containment mechanism that sequesters toxic proteins and prevents their interaction with other cellular components, thereby preserving cytoplasmic integrity (17).

However, the prolonged presence of aggresomes may disrupt cytoskeletal organization, impair intracellular trafficking, inhibit proteasome activity, and lead to organelle dysfunction, contributing to the pathogenesis of neurodegenerative diseases. Protein inclusions similar to aggresomes have been described in conditions such as Alzheimer’s disease, PD, amyotrophic lateral sclerosis, and other protein-aggregation encephalopathies (16, 18–20).

From a clinical standpoint, understanding aggresome biology facilitates the identification of novel therapeutic targets aimed at restoring proteostasis, enhancing proteasome function, activating autophagy, or preventing protein aggregation. In this context, the aggresome is not only a marker of disrupted proteostasis (20), but also a central node in the cellular defense network against structural and functional damage induced by aberrant proteins (19).

### 2.5 Inflammasome and innate signaling

The TLR4 receptor, upon recognizing saturated fatty acids or lipopolysaccharide (LPS), plays a central role in the inflammatory activation of adipocytes and immune cells such as macrophages and dendritic cells. In conditions like obesity, adipocytes generate proinflammatory metabolites that stimulate immune responses, partly through TLR4-independent pathways (13, 21). However, TLR4 is essential for the activation of NF-κB and the production of IL-1β, as well as for the activation of the NLRP3 inflammasome, a crucial component in IL-1β maturation in immune cells. DAMP-induced signaling mediated by TLR4 contributes to sterile inflammation, a non-infectious inflammatory response that can lead to tissue dysfunction. Although not directly addressed in the cited studies, TREM-1 amplifies the inflammatory signal initiated by TLR4 and can also be activated by DAMPs (14, 22). Together, TLR4 and receptors such as TREM-1 mediate inflammasome activation across different cell types, a process fundamental in inflammatory and autoimmune diseases (13, 14, 21).

Inflammasome activation has been extensively characterized in various cell types through *in vitro* protocols and murine models. In mouse macrophages, TLR agonists and TNF-α are used to induce the priming phase, followed by specific stimuli such as monosodium urate (MSU) crystals, ATP, nigericin, or cytosolic DNA to activate inflammasomes like NLRP3, NLRP1, NLRC4, and AIM2. In human peripheral blood mononuclear cells (PBMCs), the NLRP1 inflammasome can be activated using DPP8/9 inhibitors such as talabostat (15, 23). The formation of the ASC complex, or “pyroptosome”—a hallmark of inflammasome activation—is detected through immunofluorescence or protein purification methods. In murine models of peritonitis or endotoxic shock, inflammasome activation is induced by intraperitoneal injection of LPS or LPS combined with MSU, leading to IL-1β release and neutrophil infiltration. Non-canonical activation of NLRP3 by high-dose LPS has also been described. These models are essential for studying inflammasome biology, evaluating specific inhibitors, and understanding their roles in chronic inflammatory diseases (24, 25).

## 3 Emerging mechanisms of inflammasome activation

### 3.1 Endocytic trafficking and sensitization to inflammation

A recently recognized mechanism in cell biology is the activation of NLRP3 through alterations in endocytic trafficking (20). This process, essential for maintaining cellular homeostasis, involves the vesicular transport of proteins, lipids, and receptors from the plasma membrane to internal compartments such as endosomes and lysosomes (26). When disrupted—by organelle dysfunction, metabolic stress, or inflammatory stimuli—it creates a favorable environment for inflammasome activation (27).

**Figure 2:**
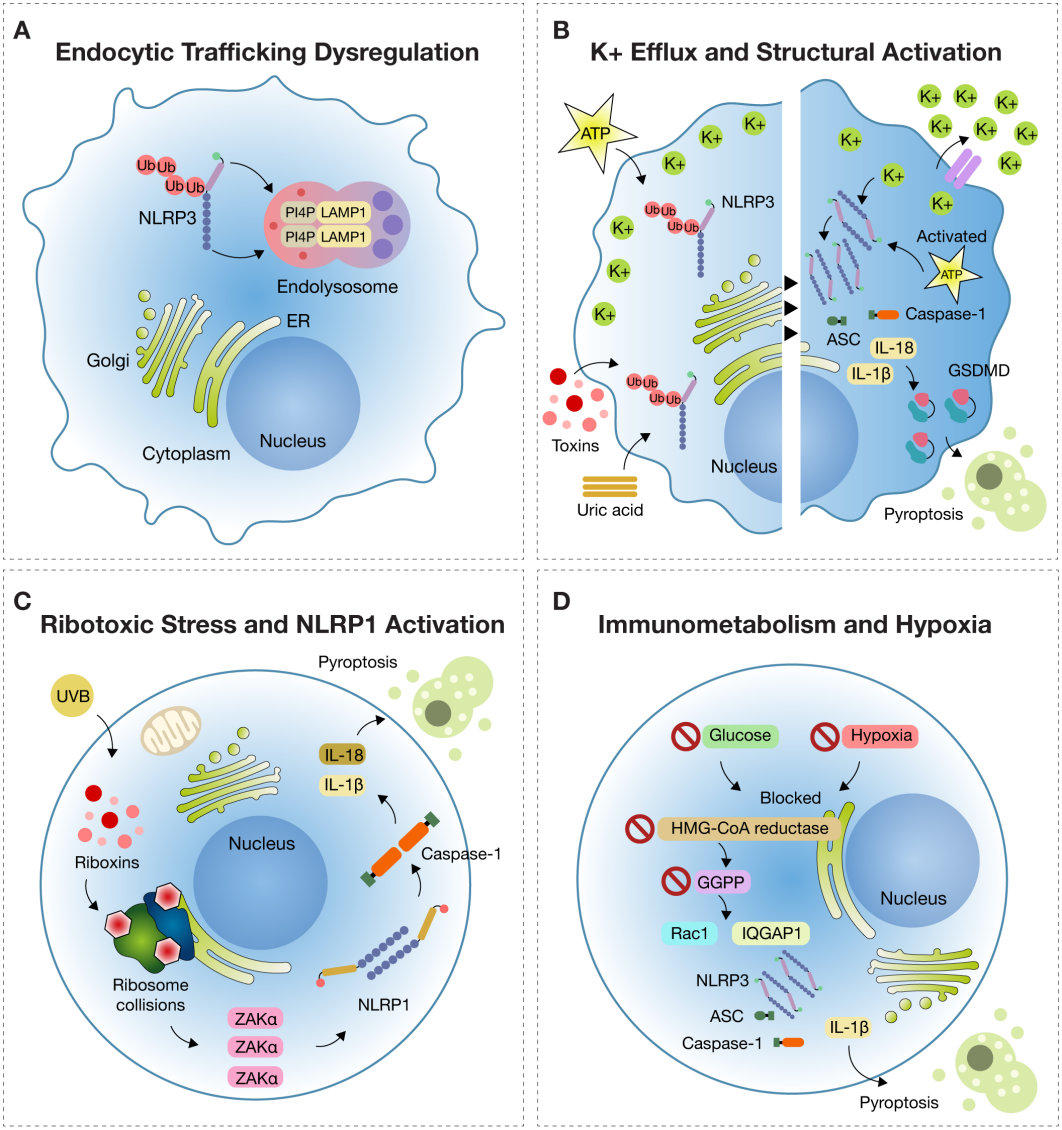
Emerging Mechanisms of Inflammasome Activation. **(A)** Endocytic trafficking dysfunction: Under disrupted endocytic trafficking, NLRP3 is recruited to endolysosomes marked by LAMP1 (lysosomal-associated membrane protein 1), where it colocalizes with PI4P (phosphatidylinositol-4-phosphate), remaining in a primed but inactive state. **(B)** Potassium efflux Potassium (K^+^) efflux triggered by stimuli such as ATP, uric acid crystals, or bacterial toxins induces NLRP3 activation by lowering intracellular K^+^, promoting ATP binding to the NACHT domain and oligomerization of NLRP3 with ASC and pro-caspase-1. This leads to IL-1β and IL-18 release and gasdermin D (GSDMD)-mediated pyroptosis. **(C)** Ribotoxic stress in NLRP1 activation: In epithelial tissues, UVB radiation and ribotoxins induce ribosome collisions sensed by the kinase ZAKα (sterile alpha motif and leucine zipper-containing kinase alpha), which triggers NLRP1 inflammasome assembly, activation of caspase-1, and pyroptosis. **(D)** Immunometabolism and Hypoxia: In metabolic stress conditions (e.g., glucose deprivation or hypoxia), inhibition of HMG-CoA reductase reduces GGPP (geranylgeranyl pyrophosphate) synthesis, causing Rac1 retention in the cytosol and binding to IQGAP1, which activates NLRP3 and drives IL-1β release and pyroptosis.

During the course of cellular dysfunction associated with inflammation, it has been observed that the NLRP3 inflammasome, initially diffusely distributed in the cytosol, is actively recruited to endolysosomal compartments. These compartments are characterized by the expression of the membrane marker lysosomal-associated membrane protein 1 (LAMP1), which indicates their lysosomal or endolysosomal identity. In these intracellular domains, NLRP3 specifically co-localizes with phosphatidylinositol-4-phosphate (PI4P), a phosphorylated lipid located on the internal membranes of the endomembrane system, particularly at the transition between late endosomes and lysosomes (27) (**Figure 2A**).

PI4P serves as a spatial anchor for NLRP3, promoting its structural stabilization and facilitating proximity to adaptor proteins such as ASC (apoptosis-associated speck-like protein containing a CARD) and pro-caspase-1—key elements for functional inflammasome assembly (27).

However, this relocalization induced by disruptions in endocytic trafficking—such as those caused by chemical inhibitors, metabolic alterations, or organelle stress—is not sufficient on its own to trigger full inflammasome activation (27). Additional danger signals, such as TLR activation or DAMP release from damaged cells, are required to trigger caspase-1 cleavage and the release of IL-1β and IL-18 (3).

This mechanism is significant because vesicular trafficking disruption, although not directly activating the inflammasome, sensitizes immune cells—lowering the threshold for activation. Subthreshold stimuli, which would not typically induce inflammation, can provoke exaggerated inflammatory responses. This sensitization is particularly relevant in chronic inflammation and metabolic diseases, where vesicular homeostasis is persistently impaired (27).

Such mechanisms help explain the persistence of inflammation in the absence of infection and open new avenues for modulating inflammation through control of intracellular trafficking and the NLRP3–PI4P interaction (28).

The consequence of this activation is the exacerbated release of IL-1β and IL-18 and the promotion of pyroptosis, contributing to tissue damage and the perpetuation of the inflammatory cycle (27). In chronic inflammatory diseases such as diabetes, atherosclerosis, chronic kidney disease, and metabolic syndromes, vesicular trafficking dysfunction is common, which could explain the sustained sterile inflammation observed in these contexts. Additionally, inflammasome activation via this mechanism may contribute to uncontrolled systemic inflammation in acute conditions such as sepsis or cytokine release syndrome (24).

This pathway represents a key point in modern inflammatory pathophysiology, opening new opportunities for the identification of specific therapeutic targets (27). Modulating endocytic trafficking, stabilizing the involved organelles, or blocking the interaction between NLRP3 and PI4P could represent innovative strategies to control inflammasome-mediated inflammatory diseases (29).

### 3.2 Potassium efflux and structural activation

Activation of the NLRP3 inflammasome is closely linked to the loss of intracellular potassium (K^+^), which acts as a critical event in initiating inflammatory signaling. Recent *in vitro* studies have shown that potassium efflux, induced by agonists such as ATP and nigericin, activates the inflammasome independently of elevated intracellular calcium (Ca²^+^) levels (30, 31). Increased cytosolic Ca²^+^ was found to be neither necessary nor sufficient to trigger the NLRP3-mediated inflammatory cascade (31, 32).

Furthermore, agonists of G protein–coupled receptors that mobilize calcium—such as the formyl peptide receptor, P2Y2 purinergic receptor, and extracellular calcium-sensing receptor—were ineffective at inducing rapid inflammasome activation compared to potassium efflux agonists. The use of BAPTA and 2-APB inhibited nigericin-induced inflammation through mechanisms unrelated to calcium signaling (31, 32).

Multiple NLRP3 activators, including oxidized apolipoproteins, have been shown to induce ionic stress and disrupt intracellular homeostasis, promoting potassium efflux and inflammasome activation in epithelial cells, including renal cells (30, 32, 33). This ionic pathway constitutes a sterile mechanism of inflammation highly relevant in metabolic, renal, and immune-mediated diseases, making its inclusion pathophysiologically justified (30, 31, 33).

The activation of the NLRP3 inflammasome represents a central immunological axis in the pathophysiology of multiple chronic inflammatory diseases. Under conditions of cellular homeostasis, NLRP3 remains in the cytosol in a closed, inactive conformation stabilized by intramolecular interactions (34). However, various sterile stimuli—such as uric acid crystals, extracellular ATP, imiquimod, or microbial toxins—induce a convergent minimal signal: the efflux of intracellular potassium, which acts as a universal trigger for complex activation (35).

Loss of intracellular K^+^ indirectly facilitates NLRP3 activation by destabilizing its autoinhibited structure and promoting a conformational transition to an open, active state. NLRP3 does not directly sense potassium efflux; rather, the resulting ionic changes alter the intracellular environment to favor inflammasome assembly. This process is mediated by two structural regions: the FISNA domain (Fish-specific NACHT-associated domain) and a flexible linker between the PYD (pyrin domain) and NACHT domains, encoded by exon 3—features unique to mammalian NLRP3. These domains facilitate repositioning of the PYD to interact with ASC, enabling the formation of the functional inflammasome (35).

BRET (bioluminescence resonance energy transfer) sensor studies have confirmed that potassium efflux leads to a sustained decrease in BRET signal, indicating a conformational opening of NLRP3. This transition exposes the nucleotide-binding pocket, allowing ATP to bind and undergo hydrolysis—an essential step for NACHT domain activation and receptor oligomerization. The resulting oligomer provides a platform for ASC nucleation and subsequent activation of pro-caspase-1, culminating in IL-1β and IL-18 maturation and pyroptosis via gasdermin D cleavage (**Figure 2B**). Understanding this structural architecture is key to advancing precision immunomodulation strategies (35).

The linker sequence (residues 92–132) organizes the helical arrangement of PYD domains within the oligomer, optimizing interaction with ASC. Its progressive deletion compromises inflammatory functionality without affecting receptor oligomerization, highlighting its role in efficient inflammasome assembly. The FISNA domain, meanwhile, acts as a structural interface for oligomerization and participates in interaction with negatively charged phospholipids such as PtdIns4P in the trans-Golgi network, especially in K^+^-independent activation contexts (e.g., imiquimod) (35).

From a clinical standpoint, the PYD-linker-FISNA region and the NACHT domain are essential for structural activation of the NLRP3 inflammasome. These regions govern the transition from resting to the active state, allowing inflammatory activation through complex formation. Pharmacologic blockade of these domains prevents IL-1β release while preserving baseline immune function (35). Moreover, recent evidence has demonstrated that oxidized mitochondrial DNA (ox-mtDNA) can directly activate the NLRP3 inflammasome independently of potassium efflux. This K^+^-independent mechanism relies on structural engagement of the pyrin domain, as shown by novel small-molecule inhibitors targeting this site (36).

MCC950 acts on the NACHT domain, preventing ATP binding and the structural opening of NLRP3. It has shown efficacy in preclinical models of inflammatory diseases such as gout, CAPS, and Alzheimer’s disease (34). Therefore, designing drugs specifically targeting these regions allows for selective immunomodulation without inducing generalized immunosuppression, offering a safe and clinically relevant therapeutic approach (35).

### 3.3 Ribotoxic stress and NLRP1 activation in epithelial immunity

The NLRP1 inflammasome constitutes a sophisticated intracellular immune surveillance system designed to detect danger signals that compromise epithelial cell integrity, particularly in the skin and respiratory tract (37). Far from being a simple molecular alarm, NLRP1 is a multiprotein complex specifically responsive to ribotoxic stress—a type of damage occurring when ribosomes, the cellular machinery responsible for protein synthesis, are blocked or impaired by ultraviolet B (UVB) radiation or certain bacterial toxins known as ribotoxins. This disruption of a vital cellular function triggers emergency inflammatory pathways (38).

The inflammatory response to ribotoxic stress is highly specialized and orchestrated by the kinase ZAKα (MAP3K20) in conjunction with the NLRP1 inflammasome, particularly in epithelial tissues. Under basal conditions, ribosomes perform uninterrupted protein translation. However, agents such as UVB radiation or microbial ribotoxins induce ribosomal collisions, thereby activating ZAKα. This kinase detects ribosomal structural stress and phosphorylates a disordered linker region (DR) of NLRP1, a domain uniquely expressed in human epithelium (38).

This phosphorylation is not a mere modification; it acts as a switch that changes the conformation of NLRP1, releasing its UPA-CARD domain, triggering internal proteolytic cleavage. This activation allows NLRP1 to assemble with the adaptor protein ASC and pro-caspase-1 into a multiprotein complex: the inflammasome (38).

Upon formation, the inflammasome activates caspase-1, a key protease that processes the inactive forms of cytokines IL-1β and IL-18 into their mature forms, promoting their extracellular release. These cytokines are potent mediators of inflammation, recruiting immune cells to the site of injury and amplifying the damage signal. Simultaneously, caspase-1 cleaves gasdermin D (GSDMD), releasing an N-terminal fragment that forms pores in the plasma membrane. This induces pyroptosis, an inflammatory form of cell death that ruptures the cell, releases DAMPs (damage-associated molecular patterns), and strengthens the immune response (38) (**Figure 2C**).

Collectively, this pathway converts an intracellular ribosomal stress signal into a robust local inflammatory response, essential for eliminating damaged epithelial cells and alerting the immune system to microbial or environmental threats. Thus, it forms a critical axis of barrier immunity.

This discovery transforms our understanding of cutaneous and respiratory inflammatory mechanisms. Sunburns, previously explained solely as radiation damage, must now also be understood as an immunological phenomenon triggered by ribosomal blockade in keratinocytes. Similarly, respiratory infections caused by bacteria that secrete ribotoxins can activate this pathway, exacerbating inflammation (38).

The NLRP1 inflammasome is activated by ribotoxic stress, such as ribosome damage or translation blockade, through proteasomal degradation of its N-terminal domain. This triggers caspase-1 activation, IL-1β and IL-18 release, and pyroptosis. Unlike NLRP3, NLRP1 senses cellular stress rather than ionic changes, offering a distinct therapeutic target. Clinically, this pathway is relevant in infections, chemotherapy toxicity, and autoinflammatory diseases. Its selective expression in epithelial tissues also enables targeted interventions in skin and mucosal inflammation. Thus, NLRP1 represents a promising focus for drug development in stress-induced inflammatory conditions (37). Furthermore, identification of checkpoints such as ZAKα and p38 opens avenues for pharmacologic intervention to prevent excessive epithelial inflammation. These insights have significant implications for treating inflammatory skin disorders, genetic photosensitivity syndromes, toxin-induced damage, and pollution-related respiratory diseases (38).

Beyond its molecular impact, this mechanism humanizes inflammation: the cell does not react to every stimulus with violence, but evaluates the type of damage, activates precise sensors, and only if the danger is real, launches a programmed response. Thus, NLRP1 becomes a rational sentinel of the epithelium, capable of balancing defense and damage (37). In an era where precision medicine is paramount, understanding and targeting this pathway offers the potential to treat inflammation while preserving essential immune functions. Even damaged ribosomes, it seems, have a voice in orchestrating immune defense (38).

### 3.4 Immunometabolism and hypoxia-induced inflammation

The activation of the NLRP3 inflammasome under conditions of energy deprivation represents a direct mechanistic bridge between cellular metabolism and innate immune responses. In human monocytes, the simultaneous restriction of glucose and oxygen—a scenario commonly observed in inflamed or ischemic tissues—initiates a specific molecular cascade: inhibition of the enzyme HMG-CoA reductase (HMGCR), which plays a central role in the mevalonate pathway. This inhibition reduces the synthesis of geranylgeranyl pyrophosphate (GGPP), a lipid molecule essential for the prenylation of small GTPases such as Rac1 (39) (**Figure 2D**).

In the absence of sufficient GGPP, Rac1 cannot undergo proper prenylation and fails to anchor to cellular membranes. Instead, it accumulates in the cytosol in its unprenylated form and binds to the scaffold protein IQGAP1. This interaction activates the NLRP3 inflammasome and leads to the release of IL-1β, a pivotal cytokine in the inflammatory response. While this mechanism is normally silenced under homeostatic conditions, it becomes pathologically overactivated in hostile microenvironments. Notably, this phenomenon is not restricted to rare genetic disorders. For example, Muckle-Wells syndrome—a CAPS caused by gain-of-function mutations in the NLRP3 gene—is characterized by exaggerated inflammasome activity, recurrent fever, urticarial rash, arthralgia, and progressive sensorineural hearing loss. However, similar inflammasome-driven processes can also be activated in common clinical contexts such as sepsis, autoimmune disorders, or hypoxic tissue injury, where metabolic stress and oxygen deprivation converge (40) (**Figure 2D**).

From a medical standpoint, this redefines inflammation as an immunometabolic phenomenon. Many intense inflammatory conditions resistant to immunomodulators may originate from intracellular metabolic control failures, rather than from external insults or classical immune dysfunction (39).

For clinicians, this means that not every inflammatory process requires direct immune suppression: it is also possible to intervene in metabolic pathways, restore prenylation, and recover cellular balance. The use of statins must be carefully evaluated; although they reduce cholesterol, they could also increase inflammation in acute hypoxic states. Likewise, selective NLRP3 inhibitors or modulators of the mevalonate pathway may represent future targeted therapies (39).

Understanding this mechanism enriches diagnostic and therapeutic reasoning across disciplines including internal medicine, critical care, rheumatology, and infectious diseases. It encourages physicians to consider that persistent fever, unresolving inflammation, or disproportionate immune responses may reflect underlying biochemical dysfunctions—and that restoring these pathways may enable inflammation to subside through the body’s intrinsic regulatory systems (39).

Statins inhibit HMG-CoA reductase, reducing the synthesis of isoprenoids like GGPP, essential for the prenylation of proteins such as Rac1. Under normal conditions, prenylated Rac1 regulates cellular functions without activating inflammation. However, in acute hypoxic states, where there is a baseline metabolic inhibition, statins may worsen this situation (39).

Unprenylated Rac1 accumulates in the cytosol, activates the NLRP3 inflammasome, and increases IL-1β release, exacerbating inflammation. Thus, in specific clinical scenarios such as sepsis or ischemic inflammation, statins may paradoxically intensify inflammatory responses. These considerations highlight the need for personalized assessment of statin therapy in patients with underlying metabolic and inflammatory dysregulation (39).

### 3.5 Pharmacologic inhibition of NLRP3 inflammasome

MCC950 and OLT1177 are selective inhibitors of the NLRP3 inflammasome, each with distinct mechanisms of action and therapeutic potential in inflammatory diseases. MCC950 directly targets the ATP-hydrolysis motif within the NACHT domain of NLRP3, blocking its activation and preventing inflammasome assembly (41–44). This inhibition suppresses the maturation of IL-1β and IL-18 without affecting other inflammasomes such as NLRC4 or AIM2, demonstrating high specificity. In contrast, OLT1177 (dapansutrile), an orally active β-sulfonyl nitrile compound (42–44), inhibits the interaction between NLRP3, ASC, and caspase-1, thereby preventing inflammasome oligomerization.

*In vitro*, OLT1177 significantly reduces the release of IL-1β and IL-18 in human macrophages and monocytes derived from patients with CAPS. It also decreases caspase-1 activity and oxidative stress, while enhancing muscle oxidative metabolism in LPS-stimulated models. *In vivo*, OLT1177 has shown protective effects in experimental autoimmune encephalomyelitis and spinal cord injury models by reducing immune cell infiltration and IL-1β levels in affected tissues. Both compounds represent promising therapeutic strategies by selectively inhibiting the NLRP3 inflammasome, a central platform in the pathogenesis of acute and chronic inflammatory conditions (**Table 1**) (41, 44).

**Table 1:** Comprehensive overview of current NLRP3 inflammasome inhibitors across human diseases.

Disease	Inflammasome Involved	Trigger/Mechanism	Downstream Effects	Therapeutic Implications
Sepsis	NLRP3	PAMPs/DAMPs; mitochondrial dysfunction; endocytic trafficking disruption	IL-1β/IL-18 release, pyroptosis, organ failure	MCC950; biomarker-based immunomodulation
Toxin-Mediated Inflammation	NLRP3	α-toxin (Clostridium septicum) induces ion efflux via pore formation	Pyroptosis, cytokine storm, vascular collapse	MCC950 blocks lethality in animal models
Parkinson’s Disease	NLRP3	Misfolded α-synuclein activates microglial inflammasome	Chronic neuroinflammation, dopaminergic neurodegeneration	MCC950 and NLRP3−/− models show neuroprotection
HIV-1 Infection	CARD8	HIV-1 protease cleaves CARD8 via NNRTI-induced premature activation	Pyroptosis of latently infected CD4+ T cells	NNRTIs repurposed for viral reservoir clearance
Respiratory Viral Infections	NLRP1	HRV 3C protease cleaves NLRP1, releasing C-terminal fragment	Caspase-1 activation, IL-1β/IL-18 secretion, epithelial pyroptosis	Targeting HRV–NLRP1 interaction in asthma/COPD
EHEC Infection (Shiga toxin)	Non-canonical (caspase-4/5)	Stx inhibits caspase-4/5-mediated GSDMD cleavage, preventing pyroptosis	Immune evasion, persistent infection, systemic inflammation	Potential restoration of pyroptosis via toxin-neutralizing approaches
Liver Regeneration	NLRP3	Hepatic macrophage activation post-hepatectomy impairs efferocytosis and repair signaling	Inflammation, impaired regeneration, reduced hepatocyte proliferation	MCC950 or NLRP3 deletion enhances regeneration, especially in steatotic livers
Metabolic Inflammation	NLRP3	MSU crystals activate inflammasome	IL-1β production, neutrophil recruitment	OLT1177 (dapansutrile) reduces IL-1β and pain in clinical trials

### 3.6 Therapeutic inflammasome modulation

The inhibition of the NLRP3 inflammasome represents a promising strategy for the treatment of acute and chronic inflammatory diseases. MCC950 is a specific inhibitor that directly targets the NACHT domain of NLRP3 by binding to the Walker B motif and blocking ATP hydrolysis, thereby preventing NLRP3 activation and inflammasome formation. This mechanism effectively suppresses the NLRP3-associated inflammatory cascade (43, 45–48).

In contrast, OLT1177 (dapansutrile) is an orally active β-sulfonyl nitrile compound that inhibits both canonical and non-canonical inflammasome activation (25, 30, 31). *In vitro*, OLT1177 reduces IL-1β and IL-18 release at nanomolar concentrations without affecting other inflammasomes such as NLRC4 or AIM2, confirming its specificity. In LPS-stimulated human blood-derived macrophages, OLT1177 decreases IL-1β levels by 60% and IL-18 by 70%. It also reduces caspase-1 activity and disrupts the formation of the NLRP3–ASC–caspase-1 complex (43, 48–50). In patients with cryopyrin-associated periodic syndromes, OLT1177 inhibits IL-1β release by up to 84%. Additionally, it lowers oxidative stress, decreases phosphorylated NF-κB levels, and enhances muscle oxidative metabolism (43, 48, 49).

In mouse models of experimental autoimmune encephalomyelitis, oral OLT1177 reduces demyelination, CD4+ T cell and macrophage infiltration, and improves clinical outcomes. In acute spinal cord injury, intraperitoneal administration protects neurological function, reduces IL-1β and IL-18 expression, and limits neutrophil and monocyte infiltration (43, 49, 51, 52). At the molecular level, OLT1177 disrupts the interaction between NLRP3 and ASC, as well as with caspase-1, preventing inflammasome oligomerization. It does not interfere with potassium efflux, gene expression, or IL-1β precursor synthesis, reinforcing its pharmacological selectivity. In healthy humans, daily doses of up to 1,000 mg for eight days produced no adverse effects or biochemical abnormalities, supporting its clinical safety. More recently, Lackner et al. described a novel small-molecule NLRP3 inhibitor that binds directly to the pyrin domain of NLRP3, effectively preventing inflammasome activation triggered by oxidized mitochondrial DNA. Unlike MCC950, which has known off-target effects, this compound suppresses IL-1β production in macrophages through a distinct and potentially safer mechanism of action, highlighting the evolving landscape of selective NLRP3-targeted therapeutics (36).

These findings position MCC950 and OLT1177 as leading inhibitors in the control of NLRP3 inflammasome activation, with potential applications in inflammatory, neurodegenerative, and autoimmune diseases, including sepsis and secondary neurological damage (**Table 1**) (43, 48, 49). In addition to these, newer inhibitors targeting alternative binding sites, such as those directed at the pyrin domain, may offer improved safety and efficacy in translational settings (36).

However, despite its promising preclinical efficacy, MCC950 has not progressed to clinical use due to safety concerns. Specifically, off-target effects, including hepatotoxicity, have been reported in animal studies, limiting its therapeutic application (53). At higher concentrations, it also inhibits other unrelated targets, raising questions about its selectivity under physiological conditions (11, 54). These limitations underscore the need for continued development of safer and more specific NLRP3 inhibitors.

### 3.7 Regulation of the inflammasome, gasdermin D, and modulation of NETs/METs

The regulation of the NLRP3 inflammasome and its downstream effector pathway via GSDMD involves a complex network of molecular mechanisms that dictate both the magnitude and duration of the inflammatory response (55, 56). This regulation is not solely dependent on activation by danger signals but also on post-translational checkpoints that control the transition from an inactive basal state to the assembly of active complexes capable of inducing pyroptosis (55, 57). Post-translational modifications such as ubiquitination, deubiquitination, and phosphorylation of specific NLRP3 domains serve as molecular switches, finely tuning the sensitivity of this inflammatory sensor (55, 58). For example, E3 ligase-mediated ubiquitination keeps NLRP3 in an inactive state, while specific deubiquitinases, such as BRCC3, facilitate its oligomerization and subsequent assembly (59, 60). Similarly, the phosphorylation of key residues can either activate or inhibit NLRP3, depending on the kinases and phosphatases involved (55, 57).

At the ionic signaling level, potassium efflux (K^+^ efflux) has emerged as a nearly universal requirement for NLRP3 activation, while chloride flux and intracellular calcium mobilization act as contextual modulators (58, 61, 62). The release of mitochondrial reactive oxygen species (mtROS) links the cellular metabolic state to inflammatory activation, integrating oxidative stress and mitochondrial dysfunction as central pathophysiological mechanisms (55, 63). Once activated, NLRP3 recruits the adaptor protein ASC, forming aggregation platforms that facilitate caspase-1 activation (64, 65). Caspase-1 then processes pro-IL-1β and pro-IL-18 into their active forms and cleaves GSDMD, releasing its N-terminal domain, which forms pores in the plasma membrane, triggering pyroptosis and the massive release of proinflammatory mediators (66, 67).

This understanding has spurred the development of selective inhibitors. MCC950 and dapansutrile (OLT1177) block the ATPase activity of NLRP3, directly impairing its assembly capacity (24, 68). Disulfiram and necrosulfonamide inhibit GSDMD oligomerization, preventing pore formation without fully suppressing upstream immune signaling (68, 69). Such agents could preserve some antimicrobial defense while minimizing the risk of severe immunosuppression (70, 71).

An increasingly recognized aspect is the interaction between inflammasome-mediated inflammation and the formation of neutrophil extracellular traps (NETs) and macrophage extracellular traps (METs) (72, 73). These structures, composed of decondensed chromatin and granular proteins, serve antimicrobial functions. However, when overproduced, they promote microvascular thrombosis, endothelial dysfunction, and organ injury. In experimental models, inhibition of peptidyl arginine deiminase 4 (PAD4) with compounds like Cl-amidine prevents histone citrullination, which is required for chromatin decondensation (74, 75), thereby blocking NET and MET formation (76–78). Similarly, recombinant DNase I degrades the extracellular DNA scaffold, reducing the viscosity of the inflammatory exudate and restoring capillary flow (79).

Colchicine, a well-known drug for gout and pericarditis, has been shown to reduce NETosis by destabilizing microtubules, interfering with neutrophil migration and activation (80). This effect, observed in clinical studies of vascular inflammation, suggests its potential repurposing as an adjunctive modulator in systemic inflammatory pathologies, such as severe sepsis or post-viral inflammatory response syndromes (81).

Combining NLRP3/GSDMD inhibitors with NET/MET modulators may reduce inflammation, microthrombosis, and multiorgan failure in sepsis and COVID-19, provided it is applied within the optimal therapeutic window, guided by biomarkers and adaptive precision clinical trials (82, 83).

## 4 Emerging therapeutic modulation strategies

### 4.1 Modulation via protein carbamylation

Activation of the NLRP3 inflammasome constitutes a key axis in the innate immune response to damage or infection signals. This process depends on the structural assembly between NLRP3 and the kinase NEK7, which triggers activation of caspase-1, maturation of cytokines IL-1β and IL-18, and cleavage of GSDMD, culminating in pyroptosis, an inflammatory form of cell death. Under physiological conditions, this mechanism eliminates pathogens and damaged cells; however, its deregulated activation may contribute to chronic inflammatory and autoimmune diseases (84). A new mechanism of negative modulation of the NLRP3 inflammasome is the specific carbamylation of lysine-593 (K593) of NLRP3, mediated by isocyanic acid, a metabolite generated endogenously by the LACC1 enzyme in inflammatory macrophages (85).

Following LPS stimulation, LACC1 catalyzes the conversion of citrulline into isocyanic acid, which diffuses into the cytosol and chemically modifies the K593 residue of NLRP3 (85). This post-translational modification interferes with the conformation of the NACHT domain of NLRP3, blocking its interaction with NEK7 and thereby inhibiting functional inflammasome assembly. This pathway does not alter gene expression of inflammatory components but acts as a molecular brake at later stages, limiting inflammatory signaling. Functional loss of LACC1 or point mutation at K593 (K593R), which prevents carbamylation, generates an exacerbated inflammatory response *in vitro* and *in vivo*, with hypersecretion of IL-1β, IL-18, and LDH, as well as greater susceptibility to murine models of sepsis and gout (84). These findings have significant clinical implications: isocyanic acid emerges as a negative metabolic regulator of the NLRP3 inflammasome, introducing a layer of immunometabolic control with therapeutic potential (86).

Consequently, this mechanism could be leveraged to develop drugs that mimic or enhance the carbamylation of NLRP3, modulating inflammation without globally suppressing the immune system—representing an innovative strategy for inflammatory diseases where NLRP3 is chronically activated (84).

### 4.2 Gasdermin redundancy and inflammatory execution

The NLRP3 pathway is essential in innate immunity against damage or infection. It activates caspase-1, which matures IL-1β and IL-18 and cleaves GSDMD to induce pyroptosis. Though once thought indispensable, it is now known that inflammation can persist without GSDMD, demonstrating alternate mechanisms within this response (87).

It has been shown that when NLRP3 is chronically activated—as in certain murine models with gain-of-function mutations (NLRP3^CA)—inflammation does not cease in the absence of GSDMD. On the contrary, animals continue to produce IL-1β and IL-18 and develop significant organ damage, especially following stimuli such as LPS or TNF-α. The key to this phenomenon lies in an alternative route: activation of caspases-8 and -3, which cleave gasdermin E (GSDME), another member of the same family. GSDME also forms pores in the membrane and enables the release of inflammatory cytokines (87). This finding reveals a molecular adaptability within the inflammasome, making it more resistant to selective pharmacologic blockade. In this context, the use of CuET, a disulfiram derivative capable of inhibiting both GSDMD and GSDME, showed significant anti-inflammatory effects (88).

Clinically, this holds great value. In autoinflammatory or neurodegenerative diseases where NLRP3 is persistently activated, inhibiting GSDMD may be only part of the treatment. If GSDME acts as an inflammatory backup, symptoms could persist despite targeted therapy. Understanding this mechanism not only provides insight into the cellular pathophysiology of inflammation but also anticipates the limits of certain treatments and encourages more comprehensive strategies (87). For physicians, this translates into the need to approach chronic inflammation with a more integrative and precise view, oriented toward the multiple arms of the immune response (88).

## 5 Scope of future studies on NLRP3 and inflammasomes

Future research on the NLRP3 inflammasome must focus on a true integration of mechanistic discoveries with clinical application (89). The primary goal is to establish well-characterized cohorts of critically ill patients—especially those with sepsis and septic shock—where longitudinal immunological profiling can be performed (90). This profiling should include quantification of plasma ASC specks, serial measurements of IL-1β and IL-18, caspase-1 activity, and, where feasible, analysis of cleaved GSDMD (64, 91). The combination of these biomarkers would allow for the stratification of patients into phenotypes with varying intensities and durations of inflammasome activation, thereby optimizing the selection process for targeted therapies (92).

Simultaneously, systems biology technologies provide powerful tools for mapping the heterogeneity of the inflammatory response (71). Single-cell transcriptomics and proteomics (scRNA-seq, scProteomics) can uncover cellular subpopulations exhibiting distinct inflammasome activation patterns, while metabolomics can link these patterns to specific energetic and oxidative states (93, 94). These approaches should be integrated with advanced imaging techniques, such as *in vivo* confocal microscopy and positron emission tomography (PET) using tracers designed to detect inflammasome components (94, 95). Direct visualization of inflammatory activity in target organs—such as the lung, kidney, or brain—could guide real-time therapeutic decision (53).

Another promising strategy is the design of adaptive clinical trials assessing selective inhibitors of NLRP3 and GSDMD, either as monotherapies or in combination with modulators of NETosis and METosis (96). These trials should not only evaluate efficacy in terms of reduced mortality or improved SOFA scores, but also long-term safety, including surveillance for late infections, chronic inflammation, or organ sequelae (48, 97). Incorporating immunological response criteria, such as the normalization of inflammasome biomarkers, would allow for dynamic therapy adjustments (48, 98).

A relatively underexplored yet clinically relevant area is the role of inflammasome activation in post-sepsis syndrome (99, 100). Preliminary evidence suggests that persistent, even low-grade, inflammasome activity may contribute to immunometabolic dysfunction, increased cardiovascular risk, and neurocognitive decline months after hospital discharge. Including inflammasome biomarkers in post-ICU follow-up protocols could help identify patients at risk and open the door for maintenance immunomodulatory interventions (101, 102).

Finally, research should broaden its focus beyond NLRP3, investigating interactions with other inflammasomes such as AIM2 and NLRC4 (55, 103, 104). In settings of mixed infections, trauma, or chronic inflammatory diseases, redundant or synergistic pathways may influence the overall inflammatory response. Comparative studies could guide the rational selection of combined or sequential therapeutic approaches (55, 104).

The key to achieving these objectives will be fostering multidisciplinary collaborations among basic scientists, clinicians, and the pharmaceutical industry (105). The development of preclinical models that accurately reproduce human immune heterogeneity—such as humanized mice or organoid models—will be crucial for predicting responses to targeted therapies. Additionally, the early inclusion of pharmacodynamic assessments and biomarker endpoints in phase I and II trials will accelerate the transition from experimental hypotheses to clinical application (24, 106).

## 6 Inflammasomes in human disease contexts

### 6.1 Sepsis and systemic inflammatory syndromes

Sepsis is a severe clinical syndrome characterized by a dysregulated systemic inflammatory response to infection, which progresses to organ dysfunction and multiorgan failure if not promptly managed (98, 107). In this process, inflammasomes emerge as key structures in the detection of danger signals and amplification of inflammation (46, 108–111). Specifically, the NLRP3 inflammasome has been recognized as a central component of innate immunity and the pathophysiology of sepsis (90).

The NLRP3 inflammasome is a cytosolic multiprotein complex activated by pathogen- or damage-associated molecular patterns (PAMPs/DAMPs), resulting in caspase-1 activation, maturation of IL-1β and IL-18, and pyroptosis through gasdermin D cleavage (46, 107–111).

Among emerging activation mechanisms, the disruption of endocytic trafficking has gained increasing attention. Under metabolic stress, infection, or organelle dysfunction, NLRP3 translocates to endolysosomal vesicles enriched in LAMP1 and phosphatidylinositol-4-phosphate (PI4P), facilitating its spatial anchoring and oligomerization. However, this relocalization alone is insufficient to induce full activation; a secondary priming signal—such as LPS or TLR agonists—is required to initiate the downstream inflammatory cascade (46, 108–111).

NLRP3 activation in sepsis represents a pivotal event in tissue injury. The massive release of IL-1β and IL-18 promotes leukocyte recruitment and amplifies the inflammatory axis. At the mitochondrial level, dysfunction and ROS release, along with cytosolic mitochondrial DNA, further enhance inflammasome activation. This proinflammatory milieu also activates necroptosis and apoptosis, contributing to the progressive failure of vital organs (112).

Clinically, understanding NLRP3 inflammasome activation provides insight into the convergence of multiple signals triggering the inflammatory storm. In this scenario, therapies targeting inflammasome components (e.g., MCC950) or modulating endocytic trafficking represent promising strategies (46, 108–111) (**Table 1**). Moreover, identifying biomarkers derived from this pathway could allow precise immunological stratification of septic patients and guide personalized therapeutic interventions (113).

### 6.2 Pyroptosis in sepsis

Inflammasome activation in sepsis, particularly through the NLRP3 complex (114–116), directly induces pyroptosis through GSDMD cleavage by caspase-11 in murine models, or caspase-4/5 in humans in response to cytosolic LPS. This non-canonical pathway leads to the formation of membrane pores (117–119), release of IL-1β and IL-18, and extensive tissue injury contributing to septic shock. Furthermore, the inflammasome can engage apoptotic cascades: caspase-1 activates BID and caspase-7, while caspase-8 is recruited following inflammasome assembly, triggering programmed apoptosis (116, 120).

Experimental models have demonstrated that lipid mediators like lysophosphatidylcholine (LPC) exacerbate inflammation by enhancing NLRP3 acetylation in macrophages through SIRT2 degradation, thereby intensifying pyroptosis (116, 120).

While pyroptosis is a lytic and proinflammatory form of cell death, apoptosis is non-lytic and immunologically silent. Nonetheless, both processes coexist in sepsis and are interconnected via inflammasome signaling. This crosstalk drives multiorgan dysfunction, epithelial barrier collapse, and the sustained hyperinflammatory state characteristic of severe sepsis (116, 119, 120).

### 6.3 CIRBP in sepsis

The cold-inducible RNA-binding protein (CIRBP) acts as a critical mediator of systemic inflammation in sepsis by integrating with inflammasome pathways. CIRBP is a stress-induced protein that, upon extracellular release, functions as a DAMP by binding to the TLR4–MD2 complex, triggering NF-κB signaling (20, 21). This activation enhances the phosphorylation of Iκκ and IκBα, increasing the stability of mRNAs encoding proinflammatory cytokines like IL-1β.

In pulmonary endothelial cells, CIRBP directly promotes NLRP3 inflammasome activation, assembly with caspase-1, IL-1β release, and pyroptosis (121, 122). This is accompanied by endothelial dysfunction, increased vascular permeability, leukocyte infiltration, and lung injury.

In hypoxic models, CIRBP also negatively regulates HIF-1α and promotes neuronal apoptosis, underscoring its dual and context-dependent role in inflammation and cell death (20, 21). Collectively, CIRBP serves as a central modulator of inflammasome-driven pathways in sepsis, linking tissue injury, RNA metabolism, and innate immunity (121, 123).

### 6.4 Toxin-mediated inflammation

The NLRP3 inflammasome is a key sensor of cytosolic danger signals and microbial toxins. In *Clostridium septicum* infections—capable of causing sepsis and gas gangrene—NLRP3 activation is triggered by α-toxin binding to glycosylphosphatidylinositol (GPI)-anchored proteins on immune cell membranes. This toxin-mediated activation has been identified as a major immunopathological mechanism in Clostridium-induced sepsis. In murine models, dysregulated inflammasome activation by α-toxin leads to rapid lethality (124–126).

From a clinical practice perspective, this mechanism offers clear therapeutic implications. The selective NLRP3 inhibitor MCC950 has shown efficacy in blocking inflammasome activation and preventing death in animal models infected with *C. septicum* (**Table 1**). This positions the inflammasome as a direct therapeutic target in severe toxin-mediated infections, where the immune response, instead of controlling the infection, contributes to its fatal outcome (124–127).

The α-toxin binds to these GPI-anchored proteins, promoting toxin oligomerization and the formation of functional pores in the plasma membrane. This process triggers the loss of critical intracellular ions such as potassium (K^+^) and magnesium (Mg²^+^), generating an ionic imbalance that acts as a danger signal for the NLRP3 inflammasome. This cytosolic sensor, highly expressed in macrophages and other myeloid cells, detects these changes and assembles into multiprotein complexes along with ASC and pro-caspase-1. Once assembled, NLRP3 activates caspase-1, promoting the cleavage of pro-IL-1β and pro-IL-18 into their mature and functional forms, as well as the activation of GSDMD, whose N-terminal domain forms additional pores in the membrane that trigger pyroptosis, an inflammatory form of cell death (124–126).

While this inflammatory cascade promotes the elimination of infected or damaged cells, excessive activation—such as that induced by α-toxin—leads to widespread tissue damage, vascular collapse, and death (124–126).

### 6.5 NLRP3 in neuroinflammation and Parkinson’s disease

PD is characterized by a progressive loss of dopaminergic neurons in the substantia nigra, associated with a chronic neuroinflammatory response. In this context, the NLRP3 inflammasome has emerged as a central immune component in the pathophysiology of dopaminergic neurodegeneration. The inflammasome activates caspase-1 in response to misfolded α-synuclein, promoting inflammation, pyroptosis, and dopaminergic neurodegeneration in PD (128, 129).

The persistent formation of the NLRP3 inflammasome in microglial cells induces a neurotoxic environment that amplifies local inflammation, oxidative stress, and synaptic dysfunction, facilitating the progressive deterioration of the nigrostriatal circuit. Overexpression of inflammasome components such as ASC and NLRP3 has been observed in both murine models and human post-mortem brain tissue with PD. Experimentally, pharmacological inhibition of NLRP3 with the compound MCC950, as well as genetic deletion of this inflammasome (NLRP3−/− mice), has shown robust neuroprotective effects, attenuating dopaminergic loss and improving motor function in 6-hydroxydopamine and PFF–α-synuclein-induced models. Immunohistochemical and Western blot confirmation of cleaved caspase-1 (p20) in these models validates the direct involvement of the inflammasome in the neurodegenerative cascade (128, 129).

In clinical practice, these findings redefine our understanding of PD not only as a neurodegenerative disorder but also as an immune-mediated disease, where inflammation represents a therapeutic target capable of modifying disease progression. NLRP3 inhibition interrupts the inflammatory cascade without fully compromising immune function, offering a promising strategy to slow or prevent PD progression. Moreover, since multiple neurodegenerative diseases share similar inflammatory pathways, this route represents a common therapeutic target in translational medicine (128, 129).

### 6.6 CARD8 inflammasome activation in HIV-1

The CARD8 inflammasome plays a fundamental role in the innate immune response to viral infections and cellular damage. This protein, which belongs to the family of innate immune sensors with a caspase recruitment domain (CARD), is activated in response to various stimuli, including those derived from HIV-1 infection. Activation begins with proteolytic cleavage of CARD8’s N-terminal by HIV-1 protease, triggering a series of inflammatory events in infected cells (130, 131).

Under normal physiological conditions, CARD8 remains inactive within cells, associated with the proteasomal complex. However, in the context of HIV-1 infection, the viral protease—initially in its unprocessed Gag-Pol polyprotein form—remains inactive. This mechanism is disrupted when non-nucleoside reverse transcriptase inhibitors (NNRTIs) induce premature activation of the viral protease, leading to CARD8 cleavage and release of a bioactive fragment known as UPA-CARD. This step is crucial, as UPA-CARD release permits inflammasome activation (130, 131).

Once activated, the UPA-CARD fragment associates with pro-caspase-1, forming an inflammatory complex that activates caspase-1. Caspase-1 cleaves proinflammatory cytokines IL-1β and IL-18, maturing them for secretion. It also processes GSDMD, whose N-terminal portion forms pores in the plasma membrane, inducing pyroptosis—a type of inflammatory cell death that eliminates infected or damaged cells (130, 131).

Pyroptosis plays an essential role in the rapid clearance of infected cells and the activation of a broader inflammatory response, crucial for pathogen defense. In the case of HIV-1, CARD8 activation and pyroptosis induction may be especially important in latent viral reservoirs. This process allows HIV-1-infected cells to be eliminated even if the virus remains quiescent in CD4+ T lymphocytes (130, 131).

Therapeutically, NNRTIs, by reactivating viral protease, opens a new strategy for eliminating latently infected cells. These inhibitors not only reactivate the viral protease but also trigger CARD8 activation, resulting in HIV-1-infected cell death via pyroptosis. This approach could help reduce residual viral load in HIV-1 patients, especially those under antiretroviral therapy (ART) who are unable to fully eliminate latent reservoirs (130, 131).

In conclusion, the CARD8 inflammasome acts as a key sensor for detecting HIV-1 protease activity, enabling the clearance of latently infected cells. The use of NNRTIs may represent a promising therapeutic strategy to induce CARD8 inflammasome activation and eradicate residual HIV-1, offering new perspectives in the functional cure of HIV.

### 6.7 NLRP1 inflammasome and viral respiratory infections

The NLRP1 inflammasome is a key protein complex in innate immunity, functioning as an intracellular sensor that detects pathogens and danger signals. NLRP1 plays a critical role in activating the inflammatory response during respiratory viral infections, such as those caused by human rhinovirus (HRV), a member of the enterovirus family. This process is triggered by a specific mechanism mediated by the HRV 3C protease, which cleaves an autoinhibitory N-terminal fragment of NLRP1, enabling its activation (132, 133).

Cleavage of the N-terminal domain of NLRP1 releases the C-terminal fragment, which facilitates inflammasome assembly. This assembly initiates caspase-1 activation, an essential protease in the inflammatory cascade. Caspase-1 activates proinflammatory cytokines such as IL-1β and IL-18, which are crucial for amplifying the immune response. In addition, GSDMD, whose N-terminal portion forms pores in the plasma membrane, inducing pyroptosis—an inflammatory form of cell death. This cell death not only eliminates infected cells but also releases signaling molecules into the extracellular space to alert the immune system and amplify the inflammatory response (132, 133).

The interaction between HRV 3C protease and human NLRP1 marks a significant advancement in understanding the molecular mechanisms underlying inflammasome activation in viral infections. At the cellular level, the process begins when the 3C protease cleaves NLRP1 specifically between amino acids Q130 and G131, releasing a functional C-terminal fragment. This fragment initiates the signaling cascade that culminates in caspase-1 activation and the subsequent inflammatory response (132, 133).

NLRP1, primarily expressed in the respiratory epithelium, is activated by the HRV 3C protease. This activation generates an inflammatory response crucial for pathogen elimination but may also exacerbate respiratory diseases such as asthma and COPD. The release of inflammatory cytokines such as IL-18 and induction of pyroptosis in epithelial cells amplify pulmonary inflammation, worsening preexisting conditions. Interrupting NLRP1 activation, either by inhibiting the viral protease or using pharmacological modulators, may represent an effective strategy to control excessive inflammation during respiratory viral infections. Targeting the HRV–NLRP1 interaction offers a promising therapeutic approach to reduce inflammatory complications, particularly in patients with chronic respiratory diseases, and may help manage exacerbations associated with these infections (132, 133).

### 6.8 Immune evasion by Shiga toxin

Shiga toxin (Stx), produced by enterohemorrhagic *Escherichia coli* (EHEC), is a phage-encoded exotoxin that acts as a potent immune evasion mechanism by interfering with the host’s inflammatory response. Under physiological conditions, the intracellular detection of cytosolic LPS by inflammatory caspases (caspase-11 in mice; caspase-4/5 in humans) triggers the non-canonical inflammasome pathway, crucial for defense against invasive Gram-negative bacteria. his pathway culminates in the activation of gasdermin D (GSDMD), pore formation in the plasma membrane, and pyroptosis—an inflammatory cell death process that eliminates infected cells and releases cytokines such as IL-1β. Shiga toxin acts as a negative modulator of the non-canonical inflammasome, directly interfering with the LPS–caspase-11–GSDMD pathway (134–136).

However, EHEC has developed an immune evasion strategy through Stx secretion, selectively blocking this response. The toxin interferes with GSDMD cleavage by activated caspase-11, preventing pore formation and thus blocking pyroptosis and the release of proinflammatory mediators. This inhibition depends on the catalytic activity of Stx and is sufficient to completely suppress non-canonical inflammasome activation, both *in vitro* and *in vivo* (134–136).

Blocking this immune pathway has significant pathophysiological consequences. First, it prevents the death of infected macrophages, allowing the pathogen to persist intracellularly. Second, it reduces the production of inflammatory cytokines essential for recruiting immune cells to the infection site, facilitating bacterial dissemination. Moreover, by suppressing pyroptosis, Stx diminishes innate immune activation, affecting the transition to an effective adaptive response. This immunosuppressive ability not only enhances bacterial virulence but also represents a pathophysiological target of interest for designing new therapeutic strategies to restore or boost innate immunity against evasive intracellular pathogens (134–136).

This immune alteration contributes to the severity of EHEC-associated diseases such as hemorrhagic colitis and hemolytic uremic syndrome. Inflammasome suppression may also explain the inefficient bacterial clearance in these infections and the increased risk of systemic complications. This mechanism may also be relevant in other clinical contexts where non-canonical inflammasome activation is essential for infection control or resolution of inflammation (134–136).

### 6.9 Liver regeneration and macrophage polarization

Although the NLRP3 inflammasome plays a central role in sterile hepatic inflammation, recent studies have revealed that it also limits liver regeneration after hepatectomy. During the initial hours following major liver resection, transient activation of NLRP3 in hepatic macrophages leads to overproduction of IL-1β and IL-18, promoting persistent inflammation that impairs hepatocyte proliferation. This effect partly results from NLRP3-mediated inhibition of efferocytosis—the clearance of apoptotic cells—through reduced expression of MerTK, a receptor critical for this function. Consequently, macrophages remain in a proinflammatory Ly6C^hi state rather than transitioning to a reparative Ly6C^lo phenotype, thereby perpetuating tissue injury rather than supporting regeneration (137).

Animal models with specific deletion of NLRP3 in myeloid cells showed improved liver regeneration, greater expression of proliferation markers such as PCNA and CCND1, and increased activity of the MerTK–HGF axis, which is critical for liver repair. Pharmacological inhibition of NLRP3 with MCC950 also enhanced regeneration in complex metabolic contexts such as hepatic steatosis. At the molecular level, these treatments restored the efferocytic capacity of macrophages, boosted their anti-inflammatory profile, and promoted an environment conducive to hepatocellular proliferation. Clinical data support these findings: in hepatectomized patients, elevated IL-1β and IL-18 levels correlate with worse liver function outcomes, suggesting that unregulated NLRP3 activity hinders recovery (137).

Targeting the structural source of inflammation in macrophages offers an opportunity for precise therapeutic intervention (89). Inhibiting NLRP3 not only reduces inflammatory damage but also enhances regenerative capacity—particularly valuable in patients with pre-existing liver disease or at high risk of postoperative liver failure (137).

## 7 NLRP3 inflammasome in COVID-19

In severe COVID-19, the hyperinflammatory state is characterized by excessive cytokine release, endothelial dysfunction, coagulopathy, and progressive tissue injury (138, 139). At the cellular level, the NLRP3 inflammasome plays a central role in driving this process. In macrophages and monocytes, SARS-CoV-2 RNA and associated cellular stressors activate the NLRP3 sensor through two sequential steps: a priming phase (140, 141), in which transcription of inflammasome components is induced, and an activation phase, where ionic fluxes, mitochondrial dysfunction, and oxidative stress converge to trigger complex assembly (82, 142).

Once assembled, NLRP3 recruits the adaptor protein ASC, enabling the activation of caspase-1. Caspase-1 cleaves pro-IL-1β and pro-IL-18 into their mature, proinflammatory forms and processes GSDMD (82, 142). The N-terminal fragment of GSDMD forms membrane pores, inducing pyroptosis, a form of lytic cell death, along with rapid cytokine release. This mechanism links intracellular sensing to systemic inflammation and organ injury (83, 143).

Recent evidence indicates that neutrophils from patients with severe COVID-19 also exhibit enhanced NLRP3 activation, further amplified by type I interferon signaling. Neutrophil pyroptosis and the formation of NETs exacerbate microvascular thrombosis and endothelial injury (144, 145). This multicellular inflammasome activity contributes to the progression of acute respiratory distress syndrome (ARDS) and the prothrombotic state observed in critical COVID-19, highlighting the intersection between innate immunity and coagulation (83, 145, 146).

Therapeutically, NLRP3 is an attractive target. Preclinical models have shown that genetic deletion of NLRP3 or pharmacologic inhibition, such as with MCC950, reduces lung inflammation in SARS-CoV-2 infection and decreases cytokine release in human monocytes (147, 148). Several clinical trials are currently evaluating NLRP3 inhibitors in severe COVID-19, aiming to suppress harmful hyperinflammation without compromising pathogen clearance (139).

Given the dual role of the inflammasome—protective in pathogen elimination but harmful when overactivated—precision immunomodulation is essential (149). This approach involves the early identification of patients with laboratory markers of excessive inflammasome activity, such as elevated IL-1β or circulating ASC specks. Timely administration of inflammasome inhibitors, potentially combined with modulators of NETosis or GSDMD, can target both intracellular triggers and extracellular amplifiers of inflammation (145, 150).

## 8 Discussion

This review exposes the inflammasome as a central immunological integrator of structural, metabolic, infectious, and environmental cues (87, 137). Rather than functioning as a binary on-off switch, NLRP3 activation operates along a dynamic continuum governed by post-translational modifications, subcellular localization, and protein–protein interactions. Regulatory mechanisms such as BRCC3-mediated deubiquitination (3, 16), and isocyanic acid-induced carbamylation of lysine-593 act as molecular rheostats, fine-tuning inflammasome activation thresholds in a context-dependent manner (108).

Beyond the molecular regulation, functionally, the inflammasome exhibits considerable plasticity. It can sustain inflammation through alternative pathways in the absence of GSDMD, with caspase-3 and caspase-8 mediating cleavage of gasdermin E (88, 107). This redundancy in inflammatory effector mechanisms underscores the evolutionary robustness of inflammasome signaling and challenges the traditional linear model of its activation (27).

Subcellular localization has emerged as another critical determinant of inflammasome activity. Its transport to the MTOC via the dynein-HDAC6 axis (26), and its colocalization with autophagy machinery suggest the presence of a spatial checkpoint regulating inflammasome assembly. This interplay with autophagy pathways presents an attractive target for therapeutic modulation—offering the possibility of attenuating pathological inflammation without broadly suppressing innate immunity, particularly relevant in chronic inflammatory and neurodegenerative conditions (1).

In infectious diseases, the inflammasome not only mediates detection of pathogen-associated molecular patterns but is also a frequent target of microbial evasion strategies (38). For example, rhinovirus-activated NLRP1 and Shiga toxin-mediated suppression of non-canonical inflammasome signaling exemplify how pathogens manipulate host immune responses to their advantage, further reinforcing the inflammasome’s centrality in host-pathogen interactions (135).

The role of inflammasomes in metabolic inflammation is increasingly evident, especially under hypoxic and nutrient-deprived conditions common to monocytes in diseases such as sepsis, hepatic steatosis, and PD. In these contexts, inflammasome activation contributes to disease progression by amplifying tissue-damaging inflammation. This pathogenic role renders the inflammasome a potential dual-purpose biomarker and therapeutic target. Agents like MCC950 and THL have demonstrated promising efficacy in preclinical models (108, 129).

Despite these advances, important knowledge gaps persist. A significant proportion of existing data arises from *in vitro* and murine models, which may not fully capture the complexity of human pathophysiology. The interactions between inflammasome activation, tissue microenvironment, and host genetic variability remain incompletely understood, highlighting the pressing need for translational research and well-designed clinical trials (90, 109). Furthermore, spatial and temporal aspects of inflammasome signaling within distinct cellular compartments are only beginning to be elucidated.

Emerging single-cell analyses have uncovered previously unrecognized heterogeneity in inflammasome activation across diverse cell types and disease contexts. This includes variations in activation thresholds, effector functions, and kinetics. For instance, microglial NLRP3 activation in PD displays distinct regulatory patterns compared to macrophage responses in sepsis, while epithelial responses to viral infections diverge from those of myeloid cells. These context-specific differences have critical implications for therapy, suggesting that effective inflammasome-targeted interventions may need to be customized at the cellular level. Technologies such as spatial transcriptomics and single-cell proteomics will be essential in mapping this heterogeneity and designing precision interventions that preserve physiological immune responses (1, 29).

Looking forward, research should aim to integrate inflammasome signaling with broader networks involving regulated cell death, metabolism, and autophagy. Understanding how these processes intersect in diseased tissues will aid in identifying novel biomarkers and tailoring personalized therapeutic approaches. Selectively modulating inflammasome components without compromising host defense mechanisms could transform treatment paradigms for a wide spectrum of inflammatory and degenerative diseases.

The evolving understanding of inflammasome architecture, regulation, and function paves the way for a more refined immunomodulatory paradigm. Rather than relying on broad-spectrum anti-inflammatory agents, future therapies may selectively inhibit pathological inflammasome activation while preserving essential immune functions. This precision-guided approach holds promise for advancing mechanistically informed, patient-specific treatments in inflammatory, infectious, and neurodegenerative diseases (109).

## 9 Conclusions

The inflammasome—particularly NLRP3—is a dynamic immune sensor that integrates structural damage, metabolic stress, and pathogen signals through tightly regulated mechanisms including post-translational modifications, intracellular trafficking, and immunometabolic cues. Its dysregulated activation contributes to a wide spectrum of inflammatory, infectious, metabolic, and neurodegenerative diseases.

Beyond host defense, inflammasomes contribute to tissue regeneration, programmed cell death, sterile inflammation, and neuroimmune regulation. Selective inhibitors—such as MCC950, THL, and OLT1177—have demonstrated efficacy in preclinical models, while emerging strategies targeting BRCC3, HDAC6, carbamylation, and trafficking pathways offer additional control points. The existence of redundant inflammatory mechanisms, such as gasdermin E–mediated pyroptosis, underscores the need for systems-level and multi-targeted approaches.

A novel direction involves engineering synthetic inflammasomes that respond only to specific pathological cues, offering potential for next-generation immunotherapies. Future research should focus on integrating inflammasome biology with the epigenome, microbiota, and neuroimmune networks, and on identifying biomarkers for personalized immunomodulation.
